# Editorial: Better Together: A Joined-Up Psychological Approach to Health, Well-Being, and Rehabilitation

**DOI:** 10.3389/fpsyg.2016.00974

**Published:** 2016-06-28

**Authors:** Donal G. Fortune, Elaine L. Kinsella, Orla M. Muldoon

**Affiliations:** ^1^Psychology, University of LimerickLimerick, Ireland; ^2^Psychology, Centre for Social Issues Research, Mary Immaculate College, University of LimerickLimerick, Ireland; ^3^Psychology, Centre for Social Issues Research, University of LimerickLimerick, Ireland

**Keywords:** health, well-being, rehabilitation, social support, social identity

It is exactly 30 years since Arthur Kleinman introduced the term “sociosomatic” in an attempt to refocus attention in the health and psychological sciences on the often apparent, yet all too frequently neglected, social aspects of illness, disorder, and well-being (Kleinman, 1986). Social and cultural causes, social mediators, and moderators, and social outcomes were suggested by Kleinman as representing an additionally helpful, legitimate, and clinically useful formulation of disorder and well-being. This framework contextualized such multifaceted intra- and inter-personal challenges within the social, cultural, and material contexts of peoples' everyday lives. Accordingly, the study of disorder and well-being necessarily requires the interdigitation of the person, their body, and their social and cultural world as essential and inter-dependent components of a comprehensive system of experience.

Since Kleinman's call to arms, one might be forgiven for perceiving psychology to have become increasingly fractionated and divided from its common or shared base. Certainly, it can be observed that professional psychology has developed into increasingly specialized and more numerous “Divisions,” further “dividing” or separating relevant and complementary knowledge bases that are likely to have increasing relevance in furthering our understanding of significant social issues. The need for such conceptual and applied integration across specialisms remains compelling, particularly in the case of health and well-being—which is the principal focus of this Frontiers Research Topic. In the initial call for papers for this Research Topic, we stated that health and well-being are best understood in terms of a combination of biological, psychological, and social factors; yet most formulations in this area remain constructed at the individual level. Indeed, it is over 10 years since Suls and Rothman observed that of all the published articles in the American Psychological Association's journal *Health Psychology*, 94% assessed psychological variables only, with minimal attention given to broader socio cultural factors (Suls and Rothman, 2004). Thus, while the biopsychosocial model is the basic explanatory approach for understanding the whole person in health and illness, the social side of the approach remains underspecified and poorly integrated.

Therefore, our principal aim as editors of this Research Topic was to encourage contributions that would permit readers to examine how social integration, social groups, social identity, and social capital influence health and well-being across a variety of outcomes and in a broad number of populations. This over-arching aim was necessarily multidisciplinary and multi-paradigmatic, and assumed equality within the contribution according to the various levels of research focus (i.e., genes, clinical services, families, peer groups, organizational groups, and so forth) across the life span. The aim was, therefore, to attempt to cross the conceptual borders between such arbitrary divisions.

As editors we attempted the challenging task of attracting and developing articles that would “stand-alone” as independent and significant contributions to the research literature, and that would also be consistent with the orientation and aims of this Research Topic, and with their companion articles.

To this end, there was a clear need to bring together interdisciplinary research that utilized a range of approaches across a number of different populations in order to better elucidate common and unique factors relevant to social integration, social groups, social identity, and social capital within an applied framework. We were particularly fortunate to receive such a high standard of contribution in the form of 15 published articles. A number of original research articles in this Research Topic include some of the more novel approaches or areas of investigation that have been informed by significant developments in social, clinical, biological, health, and occupational psychology. Other articles in this Research Topic are concerned with the application of psychological models to vulnerable populations that have been largely underrepresented in research in this area. Given that the various aspects addressed in this Research Topic interrelate in a dynamic and contingent manner, the research presented reflects a necessarily eclectic orientation and supports a breadth of social, cognitive, and physiological viewpoints across the life span.

Among the original research articles, two focussed on the limitations of unitary biomedical explanations. McInnis et al. reported that younger people who carry the oxytocin receptor gene polymorphism were more likely to engage in unhelpful coping styles to deal with negative social interactions, with resultant effects on mood. However, social support from parents and peers were fundamental in determining both coping and well-being regardless of genotype. Huber et al. reported that adolescents with cochlear implants who had additional disabilities did not significantly differ in terms of their relationships with school peers when compared with adolescents with no additional disabilities. Moreover, in an additional study, students in special schools for hearing impaired persons had more conduct problems than mainstream hearing-impaired children. This difference was partially explained by such children having greater difficulties in understanding speech in noisy backgrounds, coming from lower SES backgrounds, and single parent families (Huber et al.). No variable alone could explain comprehensively, why students in special schools have more mental health problems than mainstream pupils, however, the results reiterate the role of the social environment on mental health.

Four articles in this Research Topic assessed the relationships between identification (with an organization) and health behaviors or outcomes. Stronger social identification with an employing organization mediated the relationship between recognizing suffering of clients and burnout in carer's working with homeless adults (Ferris et al.). Moreover, Bjerregaard et al. reported that residential and community carers of older people reported more motivation when their relational identity with clients was perceived by them to be congruent with their organizational identity. Another article reported that although exposure to parental violence in the home reduces family identification generally, stronger identification with their extended family tended to be associated with lower anxiety and better self-esteem in younger people who witnessed parental violence within the home (Naughton et al.). Similarly, in a study examining burnout in sports psychologists across five countries, burnout was frequently experienced despite high levels of work engagement reported (McCormack et al.). The authors cite previous literature suggesting that high levels of work engagement and passion may buffer some of the negative effects of burnout, and in their own study report that social support appeared to facilitate recovery from burnout. Overall, our social situatedness informs our identity and our occupations in ways that directly influence our health and wellbeing (Gallagher et al.).

On the topic of identity pathways, Dingle et al. reported that, contrary to the predominant viewpoint on redemptive narratives in addiction recovery, there are other identity-related pathways leading into and out of addiction in people in recovery, specifically an identity loss and an identity gain pathway which have implications for engagement with recovery models. The authors found that socially-isolated individuals benefitted from the creation of a new valued social identity through affiliation with a therapeutic community. These findings bring attention to the idea that social factors can act as motivations for and barriers to recovery during the course of addiction.

Another theme contained within this Research Topic, concerns the importance of discriminated or discredited social identities and health outcomes. In a longitudinal study, Johnstone et al. reported that homeless people who perceived themselves to be discriminated against on the basis of their social group membership had fewer additional social group memberships at follow-up which consequently impacted their well-being (Johnstone et al.). and Kearns et al. reports that the stigma of accessing help for mental health services can mean those who identify with their organization, in this case a University, feel less able to access the services. In another article, unemployed people reported high levels of anticipated stigma which was associated with higher levels of psychological distress and increased report of physical ill-health (O'Donnell et al.).

Two articles considered social themes associated with another vulnerable population, individuals with acquired brain injury (ABI). In an interventional study with people affected by ABI, Fortune et al. found that changes in more distal social integration outcomes following rehabilitation depend upon prior attainment of positive neurodisability (i.e., physical) outcomes. Further, the authors highlight that the usual time frames used in ABI studies as follow-up may be insufficient to capture important aspects of social integration or community participation. Adopting a salutogenic perspective, Grace et al.'s meta-analysis suggested that people with ABI can experience positive identity growth, and that community and collective factors are likely to enhance posttraumatic growth experiences.

By focusing on cutting-edge research in social, clinical, biological, health, and occupational psychology, this Frontiers Research Topic allows new insights into how social integration, social support, and social identification influence health and well-being across a variety of outcomes and in a variety of populations—demonstrating that we are indeed better together. Beyond the excellent contributions that make up this Research Topic, we believe that this special focus will also give readers ideas for future research in this field, we hope, will continue to turn toward the investigation of social context in understanding wellbeing, illness, and disorder.

## Author contributions

DF: Draft outline of Editorial, planned structure and added some content. EK: Fleshed out content and summarized research articles contained within Research Topic. OM: Proofed and made minor typo amendments.

## Funding

This work was funded by the Irish Research Council New Foundations Award 2014.

### Conflict of interest statement

The authors declare that the research was conducted in the absence of any commercial or financial relationships that could be construed as a potential conflict of interest.
